# Clinical outcomes of tricuspid transcatheter edge-to-edge repair in patients with tricuspid regurgitation

**DOI:** 10.3389/fmed.2026.1839330

**Published:** 2026-05-29

**Authors:** Kuan-Chieh Tu, Cheng-Ya Lee, Jhih-Yuan Shih, Jheng-Yan Wu

**Affiliations:** 1 Division of Cardiology, Department of Internal Medicine, Chi Mei Hospital, Chiali, Taiwan; 2 Division of Cardiovascular Surgery, Department of Surgery, Chi Mei Medical Center, Tainan, Taiwan; 3 Division of Cardiology, Department of Internal Medicine, Chi Mei Medical Center, Tainan, Taiwan; 4 Department of Public Health, College of Medicine, National Cheng Kung University, Tainan, Taiwan; 5 Department of Nutrition, Chi Mei Medical Center, Tainan, Taiwan

**Keywords:** heart failure, major adverse kidney events, mortality, transcatheter edge-to-edge repair, tricuspid regurgitation

## Abstract

**Objective:**

To evaluate the association between tricuspid transcatheter edge-to-edge repair (T-TEER) and long-term clinical outcomes in patients with tricuspid regurgitation (TR) compared with medical therapy alone.

**Methods:**

We conducted a retrospective cohort study using the TriNetX global federated health research network. Adults with TR between 2015 and 2025 were identified and categorized into T-TEER and non-procedure groups. Propensity score matching was performed to balance baseline characteristics. The primary outcome was all-cause mortality. Secondary outcomes included heart failure exacerbation, emergency department visits, hospitalization, and major adverse kidney events (MAKEs). Cox proportional hazards models were used to estimate hazard ratios (HRs) with 95% confidence intervals (CIs).

**Results:**

After matching, 483 patients were included in each group. T-TEER was associated with a significantly lower risk of all-cause mortality at 1 year (HR 0.59, 95% CI 0.41–0.86), with sustained benefit at 3 years (HR 0.65, 95% CI 0.48–0.88) and 5 years (HR 0.68, 95% CI 0.52–0.91). T-TEER was also associated with reduced risks of heart failure exacerbation (HR 0.51), hospitalization (HR 0.60), emergency visits (HR 0.63), and MAKEs (HR 0.60) at 1 year. These associations remained consistent across longer follow-up.

**Conclusion:**

In this large real-world cohort, T-TEER was associated with lower risks of mortality and adverse clinical outcomes compared with medical therapy alone. These findings support the potential prognostic benefit of T-TEER in patients with TR.

## Introduction

Historically, the tricuspid valve (TV) has been termed the “forgotten valve” despite being the largest cardiac valve (1). Its characterization has been hindered by a complex anatomical structure and dynamic physiology. Tricuspid regurgitation (TR) occurs when leaflet coaptation is compromised by various etiologies. Primary (organic) TR, caused by intrinsic abnormalities of the valvular apparatus—such as infective endocarditis, carcinoid syndrome, or myxomatous degeneration leading to prolapse or flail leaflets—accounts for a minority of cases. Conversely, TR is predominantly secondary (functional) in origin, resulting from left-sided heart pathologies (e.g., heart failure [HF]), pulmonary hypertension, or right ventricular dysfunction (e.g., following right ventricular myocardial infarction) (2).

Severe TR is independently associated with increased mortality and morbidity (3). Historically, isolated tricuspid valve surgery has been associated with suboptimal outcomes, largely due to late-stage referral and advanced right ventricular dysfunction at the time of intervention (4, 5). According to current American Heart Association (AHA) guidelines, Class I indications for severe TR intervention remain primarily limited to patients undergoing concomitant left-sided heart surgery, while the European Society of Cardiology (ESC) similarly recommends surgery mainly in patients undergoing left-sided valve surgery or in carefully selected low-risk cases, discouraging intervention in those with advanced right ventricular dysfunction or severe pulmonary hypertension (6, 7). These guideline frameworks reflect a traditional surgical paradigm; however, they also underscore the importance of earlier intervention before irreversible right ventricular remodeling occurs, thereby highlighting the evolving and expanding role of transcatheter therapies in the management of severe TR.

Transcatheter tricuspid valve interventions have rapidly emerged as an important therapeutic option for patients with severe TR who are at high surgical risk (7). Among available therapies, T-TEER remains the most widely adopted approach because of its favorable safety profile and growing evidence supporting symptomatic and functional improvement. However, anatomical suitability is critical for procedural success, as large coaptation gaps, severe leaflet tethering, and torrential TR may limit the effectiveness of T-TEER and favor consideration of transcatheter tricuspid valve replacement (TTVR). Recent reviews have suggested that TTVR may provide more complete TR elimination in anatomically complex cases, whereas T-TEER may be better tolerated in patients with advanced right ventricular dysfunction because of its less abrupt hemodynamic changes (8).

T-TEER enhances valvular competence by approximating the leaflets and exerting an indirect annuloplasty effect. Emerging studies support the safety of T-TEER, demonstrating significant reductions in TR severity and improvements in quality of life (QoL) (9–12). The TRILUMINATE Pivotal trial reported a superior composite endpoint outcome (all-cause mortality, TV surgery, HF hospitalization, and QoL improvement) in the intervention group, driven primarily by QoL benefits. Furthermore, reduced HF hospitalization rates were observed in the intervention arm at the two-year follow-up (13). Similarly, the TRI-FR trial demonstrated that T-TEER could effectively reduce TR severity and improve patient-reported outcome measures (PROMs) (14). However, large-scale real-world data regarding the comparative efficacy and safety of T-TEER versus medical therapy remain limited. To address this knowledge gap, we conducted a retrospective cohort study using the TriNetX Research Network—comprising 48 healthcare organizations (HCOs) across the United States—to compare clinical outcomes between T-TEER and medical management in patients with TR.

## Methods

### Data source

This retrospective cohort study was conducted using data from TriNetX, a global federated health research network that integrates electronic health records from approximately 171 million patients worldwide. The database contains extensive clinical information, including diagnostic and procedural codes, medication prescriptions, laboratory measurements, and genomic data. TriNetX provides secure and real-time access to de-identified, aggregated datasets derived from a demographically and geographically diverse population across hospitals, primary care facilities, and specialty care institutions. The platform operates under a waiver approved by the Western Institutional Review Board because only aggregate statistical results are accessible and no individual-level identifiers are available. The study was performed in accordance with the Strengthening the Reporting of Observational Studies in Epidemiology (STROBE) guidelines.

### Study design

Adult patients aged 18 years or older with a documented diagnosis of TR were identified using ICD-10-CM codes I36.1, I36.8, or I36.9 between January 1, 2015, and November 30, 2025. The study period began in 2015 because ICD-10 procedural codes for T-TEER became available only after implementation of the ICD-10 coding system that year. Eligible patients were categorized into two groups. Patients who underwent T-TEER within 3 years after the TR diagnosis were assigned to the T-TEER group, whereas those without a record of T-TEER intervention were assigned to the control group. T-TEER procedures were identified using the ICD-10-PCS code 02UJ3JZ. The index date was defined as the date of the first recorded T-TEER procedure for patients in the T-TEER group and as the date of TR diagnosis for patients in the control group. Patients who had previously undergone procedures classified under heart and great vessels repair involving the TV before the index date were excluded. In addition, individuals with documented study outcomes prior to the index date were excluded from the analysis (15).

### Covariates and propensity score matching

Based on the predefined cohort definitions, index dates, outcome measures, and potential confounders, a covariate matrix was constructed using patient-level information collected during the 12 months preceding the index date. Propensity scores were estimated through logistic regression to model the probability of receiving the comparator treatment conditional on baseline characteristics. One-to-one nearest neighbor matching was performed using a greedy matching algorithm, with a caliper width defined as 0.1 times the pooled standard deviation of the logit of the propensity score. Patients in the smaller treatment group were matched to the most comparable individuals in the larger group. Covariate balance between the matched cohorts was assessed using standardized mean differences, with a value below 0.1 considered indicative of adequate balance. Matching was conducted without replacement, and all outcome analyses were restricted to the propensity score matched cohorts to minimize potential imbalance introduced by unmatched participants.

TR severity was unavailable in the database; therefore, patients could not be stratified by echocardiographic grading, and propensity matching was performed using available clinical surrogates related to disease severity. The propensity score matching (PSM) model incorporated a comprehensive set of baseline characteristics to enhance comparability between treatment groups. Demographic variables included age at the index date, reported as mean (SD), sex categorized as female or male, and race categorized as White, Black or African American, Asian, or unknown. Clinical comorbidities included diabetes mellitus, hypertension, dyslipidemia, secondary pulmonary hypertension, chronic kidney disease, paroxysmal atrial fibrillation, edema not elsewhere classified, right heart failure, presence of a prosthetic heart valve, chronic obstructive pulmonary disease, presence of a cardiac pacemaker, other diseases of the liver, biventricular heart failure, ascites, peripheral vascular diseases, presence of a xenogenic heart valve, presence of an automatic implantable cardiac defibrillator, presence of other heart valve replacement, and cerebral infarction. Procedural variables included coronary artery bypass and coronary artery dilation. Baseline renal function was assessed using estimated glomerular filtration rate (eGFR), reported as mean (SD) in mL/min/1.73 m^2^ and additionally categorized as <45 mL/min/1.73 m^2^, a clinically relevant threshold commonly used to identify moderate-to-severe chronic kidney disease. Body mass index (BMI) was incorporated as a continuous variable reported as mean (SD) in kg/m^2^ and as a categorical variable defined as ≥30 kg/m^2^, based on the standard clinical definition of obesity. Natriuretic peptide B (BNP) levels were additionally categorized using a threshold of ≥100 pg/mL, consistent with clinically established thresholds commonly used in HF evaluation.

### Outcomes and follow-up

The primary outcome was all-cause mortality. Secondary outcomes included HF exacerbation, all-cause emergency department visits, all-cause hospitalization, and major adverse kidney events (MAKEs), defined as a composite renal outcome including acute kidney injury, end-stage renal disease, dependence on dialysis, or kidney transplant status identified using ICD-10-CM diagnostic codes. Follow-up began 1 month after the index date and continued until the earliest occurrence of an outcome event, the date of the last recorded clinical encounter, death, or the end of the predefined follow-up periods at 1 year, 3 years, and 5 years after the index date, whichever occurred first.

### Statistical analysis

Continuous variables were summarized as mean values with SDs, whereas categorical variables were expressed as counts with corresponding percentages. To reduce potential confounding and improve baseline comparability between treatment groups, PSM was performed before conducting the primary and subgroup analyses. Time to event outcomes were analyzed using Cox proportional hazards regression models to estimate hazard ratios (HRs) with 95% confidence intervals (CIs). Event free survival between groups was evaluated using Kaplan Meier survival analysis, and group differences were assessed using log rank tests.

To assess the robustness of the observed associations, E values were calculated to estimate the minimum strength of association that an unmeasured confounder would require with both the exposure and the outcome to account for the observed effect estimates (16). All statistical analyses were conducted using the TriNetX analytics platform.

## Results

**Figure 1:**
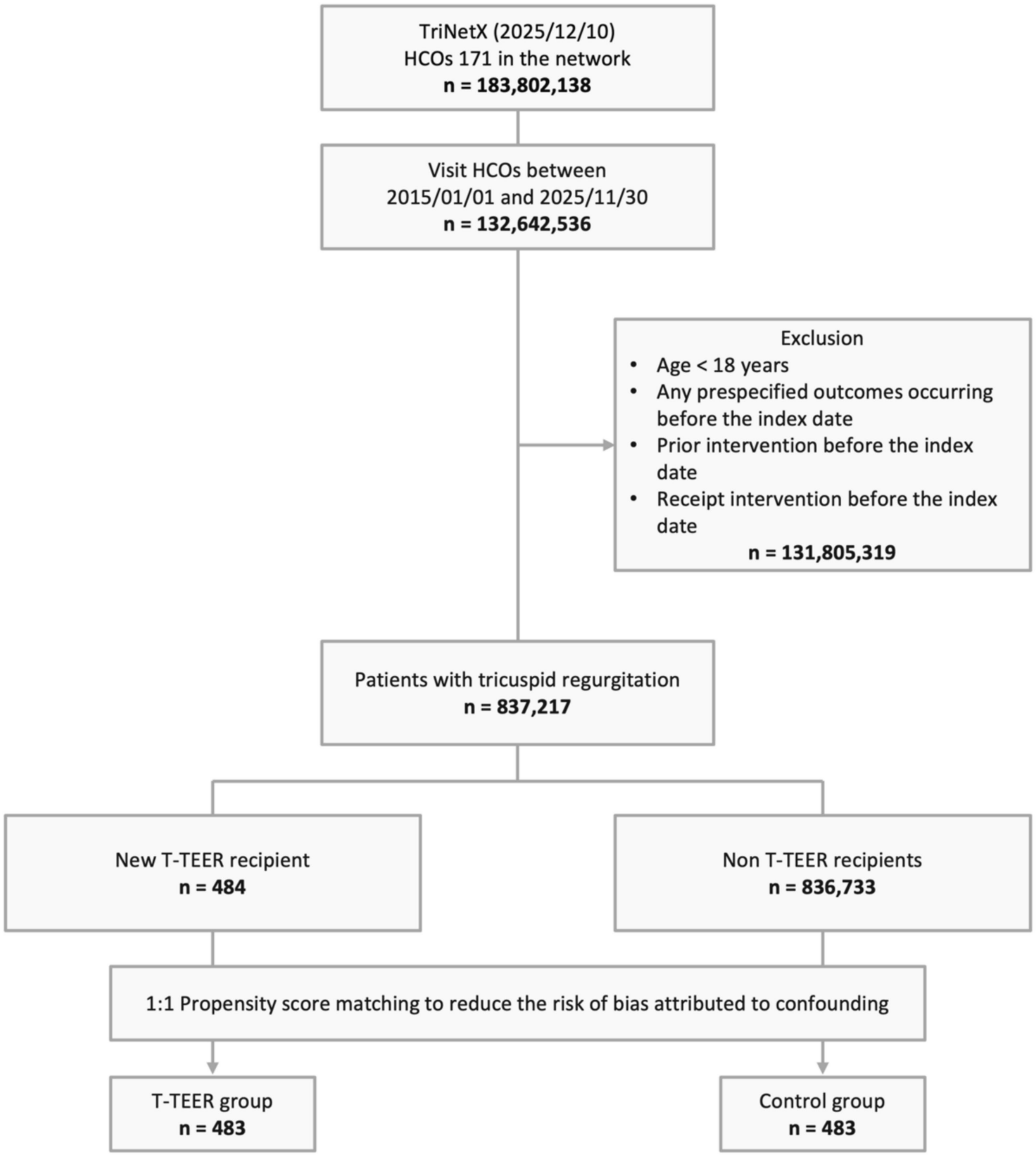
Study design and selection flow. HCOs, health care organizations; T-TEER, tricuspid transcatheter edge-to-edge repair.

After excluding younger patients according to the predefined age criteria, a total of 484 patients who underwent T-TEER were included in the study. Additionally, 836,733 patients with a diagnosis of TR were identified and included as the comparator cohort (Figure 1).

Overall, the majority of the included patients were White and African American. Prior to PSM, patients in the T-TEER group were older, had a lower proportion of African American patients, and exhibited a higher prevalence of dyslipidemia, hypertension, diabetes mellitus, atrial fibrillation or flutter, peripheral vascular disease, chronic kidney disease, liver disease, lung disease, autoimmune disease, implantable cardiac devices, biventricular heart failure, right-sided heart failure, prior cardiac surgery, edema, ascites, paroxysmal atrial fibrillation, and secondary pulmonary hypertension.

After PSM, 483 patients were matched in each group, with no significant differences observed in baseline characteristics between the two cohorts. These matched cohorts were subsequently included in the outcome analyses. Detailed baseline characteristics are presented in Table 1. The median follow-up duration was 671 days.

**Table 1:** Baseline characteristics of included subjects.

Variables	Before matching			After matching		
	T-TEER group (*n* = 484)	Control group (*n* = 836,733)	Std. diff.	T-TEER group (*n* = 483)	Control group (*n* = 483)	Std. diff.
Age at index, years						
Mean (SD)	77.2 (10.7)	65.6 (16.8)	0.826	77.2 (10.7)	76.8 (11.1)	0.033
Sex, *n* (%)						
Female	281 (58.1)	462,514 (55.3)	0.055	280 (58)	277 (57.4)	0.013
Male	201 (41.5)	373,473 (44.7)	0.063	201 (41.6)	203 (42)	0.008
Race, *n* (%)						
White	355 (73.3)	590,176 (70.6)	0.061	354 (73.3)	350 (72.5)	0.019
Black or African American	48 (9.9)	125,322 (15)	0.154	48 (9.9)	47 (9.7)	0.007
Asian	20 (4.1)	39,642 (4.7)	0.03	20 (4.1)	16 (3.3)	0.044
Unknown race	43 (8.9)	59,684 (7.1)	0.064	43 (8.9)	51 (10.6)	0.056
Comorbidities, *n* (%)						
Diabetes mellitus	145 (30)	188,640 (22.6)	0.169	145 (30)	154 (31.9)	0.04
Hypertension	325 (67.1)	432,686 (51.8)	0.318	324 (67.1)	337 (69.8)	0.058
Dyslipidemia	325 (67.1)	375,828 (45)	0.459	324 (67.1)	335 (69.4)	0.049
Secondary pulmonary hypertension	286 (59.1)	77,595 (9.3)	1.234	285 (59)	312 (64.6)	0.098
Chronic kidney disease	229 (47.3)	152,568 (18.2)	0.651	228 (47.2)	234 (48.4)	0.025
Paroxysmal atrial fibrillation	202 (41.7)	101,339 (12.1)	0.708	201 (41.6)	204 (42.2)	0.013
Edema, not elsewhere classified	127 (26.2)	91,721 (11)	0.4	127 (26.3)	118 (24.4)	0.043
Right heart failure	125 (25.8)	9,640 (1.2)	0.775	124 (25.7)	103 (21.3)	0.099
Presence of prosthetic heart valve	115 (23.8)	24,729 (3)	0.642	114 (23.6)	114 (23.6)	0
Chronic obstructive pulmonary disease	105 (21.7)	82,404 (9.9)	0.329	105 (21.7)	113 (23.4)	0.04
Presence of cardiac pacemaker	101 (20.9)	36,502 (4.4)	0.513	100 (20.7)	104 (21.5)	0.02
Other diseases of liver	88 (18.2)	50,948 (6.1)	0.377	88 (18.2)	107 (22.2)	0.098
Biventricular heart failure	62 (12.8)	6,098 (0.7)	0.495	62 (12.8)	49 (10.1)	0.084
Ascites	60 (12.4)	22,849 (2.7)	0.372	60 (12.4)	65 (13.5)	0.031
Peripheral vascular diseases	58 (12)	50,051 (6)	0.211	57 (11.8)	62 (12.8)	0.032
Presence of xenogenic heart valve	52 (10.7)	7,151 (0.9)	0.433	51 (10.6)	49 (10.1)	0.014
Presence of automatic (implantable) cardiac defibrillator	35 (7.2)	21,122 (2.5)	0.22	34 (7)	35 (7.2)	0.008
Presence of other heart-valve replacement	34 (7)	4,408 (0.5)	0.346	33 (6.8)	25 (5.2)	0.07
Cerebral infarction	25 (5.2)	44,377 (5.3)	0.006	25 (5.2)	36 (7.5)	0.094
Procedure, *n* (%)						
Bypass coronary artery	10 (2.1)	3,585 (0.4)	0.148	10 (2.1)	10 (2.1)	0
Dilation of coronary artery	10 (2.1)	1,435 (0.2)	0.181	10 (2.1)	10 (2.1)	0
eGFR, mL/min/1.73 m^2^						
Mean (SD)	50.4 (25.3)	70 (32.9)	0.67	50.4 (25.3)	51.5 (28.5)	0.041
<45	263 (54.3)	158,918 (19)	0.788	262 (54.2)	276 (57.1)	0.058
Natriuretic peptide B, pg/mL						
Mean (SD)	737.4 (895.4)	1,060.9 (3,457.5)	0.128	738 (898.2)	1,920.8 (4,111.6)	0.397
≥100	148 (30.6)	65,291 (7.8)	0.604	147 (30.4)	151 (31.3)	0.018
Body mass index, kg/m^2^						
Mean (SD)	26.2 (5.6)	28.7 (7.2)	0.397	26.2 (5.6)	28.1 (7.1)	0.298
≥30	159 (32.9)	250,413 (30)	0.063	158 (32.7)	170 (35.2)	0.052

eGFR, estimated glomerular filtration rate; Std. Diff., standardized difference; T-TEER, tricuspid transcatheter edge-to-edge repair. Standardized difference (std. diff.) < 0.1 is considered a small difference.

### Primary outcome

**Figure 2:**
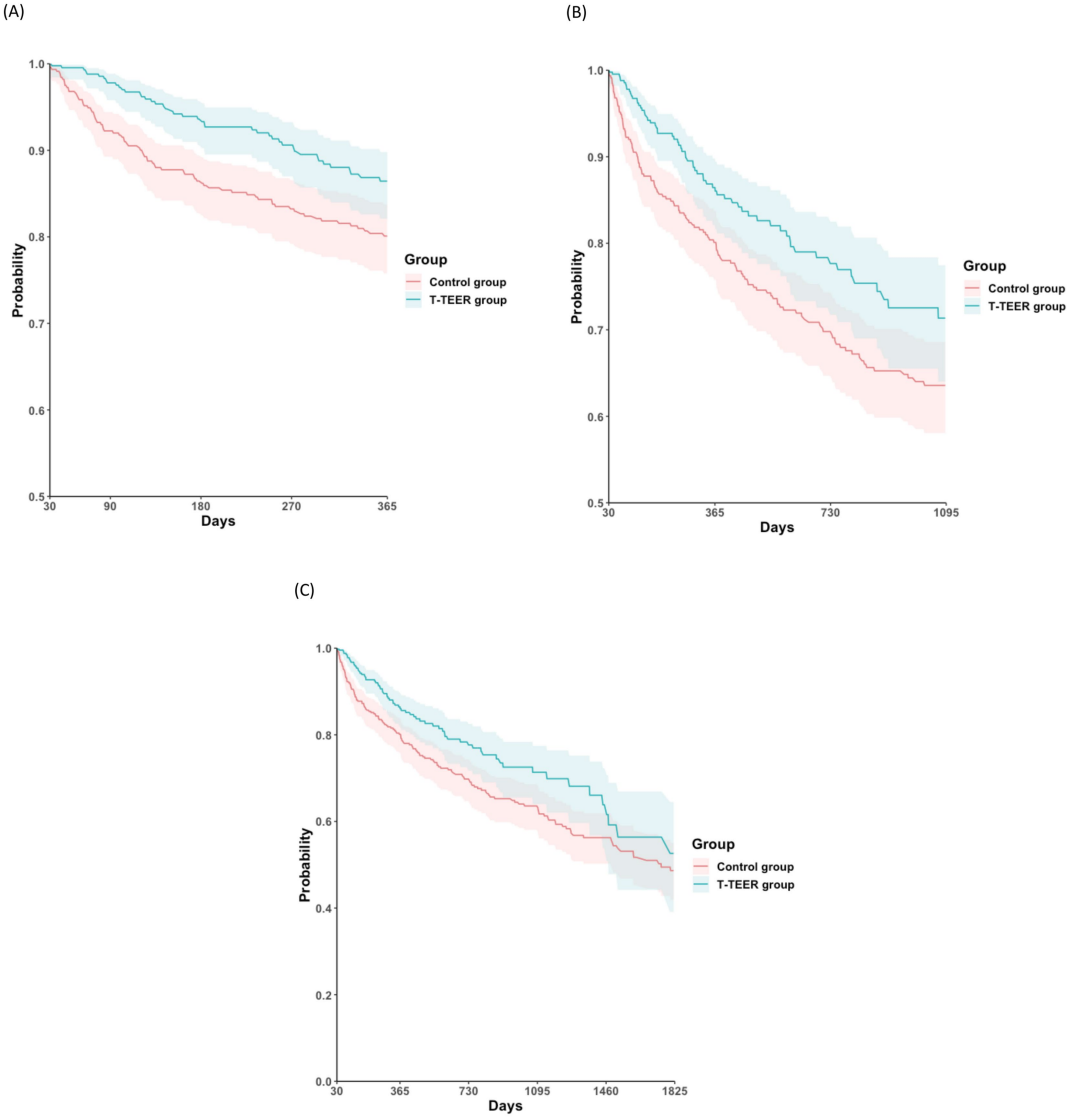
Kaplan–Meier time-to-event free curves for all-cause mortality from 1 month after the index date through **(A)** 1-year; **(B)** 3-year; **(C)** 5-year. T-TEER, tricuspid transcatheter edge-to-edge repair.

The Table 2 summarize the clinical outcomes between the two groups. During follow-up, 44 patients (9.1%) in the T-TEER group and 79 patients (16.4%) in the non-procedure group died. The T-TEER group was associated with a significantly lower 1-year all-cause mortality compared with the non-procedure group (HR, 0.59; 95% CI, 0.41–0.86; *p* = 0.005; Table 2 and Figure 2A). Temporal analyses demonstrated that the survival benefit associated with T-TEER persisted at 3 years (HR, 0.65; 95% CI, 0.48–0.88; *p* = 0.005; Table 3 and Figure 2B) and 5 years (HR, 0.68; 95% CI, 0.52–0.91; *p* = 0.008; Table 4 and Figure 2C). The observed associations were accompanied by moderate E-values ranging from 2.3 to 2.8, suggesting a degree of robustness against potential unmeasured confounding.

**Table 2:** Hazard ratios of outcomes between the T-TEER and the control groups from 1-month to 1-year follow-up after the index date.

Outcome	T-TEER group (*n* = 483)		Control group (*n* = 483)		HR (95% CI)	*p* value	*E*-value (95% LCL)
	Events (*n*)	Incidence rate (%)	Events (*n*)	Incidence rate (%)			
Primary outcome							
All-cause mortality	44	9.1	79	16.4	0.59 (0.41,0.89)	0.005	2.8 (1.5)
Secondary outcomes							
Heart failure exacerbation	58	12.0	112	23.2	0.51 (0.37,0.70)	<0.001	3.3 (2.2)
All-cause ED visit	90	18.6	140	29.0	0.63 (0.48,0.82)	<0.001	2.6 (1.7)
All-cause hospitalization	152	31.5	227	47.0	0.60 (0.49,0.74)	<0.001	2.7 (2.0)
MAKEs	89	18.4	143	29.6	0.60 (0.46,0.79)	<0.001	2.7 (1.9)

CI, confidence interval; HR, hazard ratio; LCL, lower confidence limit; MAKEs, major adverse kidney events; T-TEER, tricuspid transcatheter edge-to-edge repair.

**Table 3:** Hazard ratios of outcomes between the T-TEER and the control groups from 1-month to 3-year follow-up after the index date.

Outcome	T-TEER group (*n* = 483)		Control group (*n* = 483)		HR (95% CI)	*p* value	*E*-value (95% LCL)
	Events (*n*)	Incidence rate (%)	Events (*n*)	Incidence rate (%)			
Primary outcome							
All-cause mortality	67	13.9	127	26.3	0.65 (0.48,0.88)	0.005	2.5 (1.5)
Secondary outcomes							
Heart failure exacerbation	79	16.4	148	30.6	0.59 (0.45,0.77)	<0.001	2.8 (1.9)
All-cause ED visit	116	24.0	189	39.1	0.65 (0.52,0.82)	<0.001	2.5 (1.7)
All-cause hospitalization	179	37.1	266	55.1	0.63 (0.52,0.76)	<0.001	2.6 (2.0)
MAKEs	110	22.8	185	38.3	0.62 (0.49,0.79)	<0.001	2.6 (1.9)

CI, confidence interval; HR, hazard ratio; LCL, lower confidence limit; MAKEs, major adverse kidney events; T-TEER, tricuspid transcatheter edge-to-edge repair.

**Table 4:** Hazard ratios of outcomes between the T-TEER and the control groups from 1-month to 5-year follow-up after the index date.

Outcome	T-TEER group (*n* = 483)		Control group (*n* = 483)		HR (95% CI)	*p* value	*E*-value (95% LCL)
	Events (*n*)	Incidence rate (%)	Events (*n*)	Incidence rate (%)			
Primary outcome							
All-cause mortality	75	15.5	153	31.7	0.68 (0.52,0.91)	0.008	2.3 (1.4)
Secondary outcomes							
Heart failure exacerbation	82	17.0	162	33.5	0.59 (0.45,0.77)	<0.001	2.8 (1.9)
All-cause ED visit	122	25.3	203	42.0	0.66 (0.53,0.83)	<0.001	2.4 (1.7)
All-cause hospitalization	185	38.3	275	56.9	0.64 (0.53,0.78)	<0.001	2.5 (1.9)
MAKEs	115	23.8	198	41.0	0.63 (0.50,0.80)	<0.001	2.6 (1.8)

CI, confidence interval; HR, hazard ratio; LCL, lower confidence limit; MAKEs, major adverse kidney events; T-TEER, tricuspid transcatheter edge-to-edge repair.

### Secondary outcome

The T-TEER group was associated with lower rates of HF exacerbation, emergency department visits, and all-cause hospitalization during the 1-year follow-up period.

Within 1 year, HF exacerbation occurred in 58 patients (12.0%) in the T-TEER group and 112 patients (23.1%) in the non-procedure group (HR, 0.51; 95%CI, 0.37–0.70, *p* < 0.001). Emergency department visits were observed in 90 patients (18.6%) in the T-TEER group and 140 patients (29.0%) in the non-procedure group (HR, 0.63; 95% CI, 0.48–0.82, *p* < 0.001). All-cause hospitalization occurred in 152 patients (31.5%) in the T-TEER group, representing a significantly lower risk compared with the non-procedure group (HR, 0.60; 95% CI, 0.49–0.74, *p* < 0.001). In addition, T-TEER was associated with a more favorable composite renal outcome during the first year of follow-up (HR, 0.60; 95% CI, 0.46–0.79, *p* < 0.001). These associations remained consistent at 3-year and 5-year follow-up.

## Discussion

In this large-scale, real-world cohort study, we demonstrated that T-TEER was associated with a superior survival profile compared with medical management alone in patients with significant TR. Following PSM to mitigate baseline imbalances, T-TEER was linked to a 41% relative risk reduction in 1-year all-cause mortality, a benefit that remained robust at the 3- and 5-year marks. Beyond survival, T-TEER was consistently associated with lower rates of HF exacerbation, emergency department visits, and all-cause hospitalizations, along with a more favorable renal composite outcome. These findings suggest that T-TEER may provide clinical benefits in a high-risk population traditionally managed conservatively, potentially extending its utility beyond symptomatic palliation alone.

The survival advantage observed in our study aligns with the growing body of evidence supporting transcatheter interventions for TR, yet provides novel longitudinal insights. While recent randomized controlled trials (RCTs) have confirmed the efficacy of T-TEER in reducing TR severity and improving QoL, their impact on long-term mortality has remained modest or neutral (9, 13, 14). TRILUMINATE pivotal trial reported 2-year mortality rates of 17–18%, with no statistically significant difference from medical therapy (13). In contrast, our intervention cohort demonstrated lower mortality rates (9.1% at 1 year; 13.9% at 3 years) compared with those reported in prior RCT (9). Several factors may account for this discrepancy. Differences in baseline risk profile, earlier timing of intervention, procedural selection in real-world practice, and residual confounding despite matching may have contributed to the more favorable outcomes observed in our cohort. Nevertheless, the progressive separation of survival curves over time suggests that effective TR reduction may confer not only symptomatic improvement but also durable prognostic benefit. Our findings extend the existing literature by demonstrating an association between T-TEER and favorable long-term survival up to 5 years in a large, matched cohort, supporting the hypothesis that effective reduction of TR may influence the progression of right-sided HF beyond symptomatic improvement alone.

T-TEER was associated with lower rates of HF exacerbation, emergency department visits, and all-cause hospitalization within the first year, with these associations remaining evident during longer-term follow-up. These findings are consistent with randomized evidence from the TRILUMINATE Pivotal Trial, which demonstrated significant reductions in HF hospitalizations following T-TEER (9, 13). Similarly, the TRI-FR Trial showed reduced HF events in patients undergoing T-TEER compared with medical therapy alone (14). Our study also demonstrated a reduction in major adverse kidney events in the T-TEER group, observed in both short- and long-term follow-up. Prior studies have suggested that successful T-TEER may be associated with favorable changes in renal function; however, the magnitude of improvement was modest when compared with medical therapy alone (17). Several mechanisms may explain the observed benefit. Severe TR leads to chronic volume overload of the right ventricle (RV), progressive RV dilation and dysfunction, hepatic congestion, renal impairment, and recurrent HF admissions (18–20). By reducing regurgitant volume, T-TEER likely decreases RV wall stress, improves forward cardiac output, and alleviates systemic venous congestion (21). The lower rates of HF exacerbations and hospitalizations observed in our study are consistent with this hemodynamic rationale. Moreover, the favorable renal composite outcome may suggest that congestion relief could help mitigate cardiorenal syndrome, a major contributor to adverse prognosis in TR (22, 23). The persistence of these associations across 1-, 3-, and 5-year follow-up further supports the hypothesis that earlier mechanical correction may help slow the progression of right-sided HF.

The relatively small number of patients undergoing T-TEER despite the large population diagnosed with TR likely reflects the recent adoption of transcatheter tricuspid interventions in clinical practice. In addition, T-TEER is currently performed mainly in selected high-risk patients at experienced centers, and patient eligibility is often limited by anatomical suitability, disease severity, comorbidities, and device availability. Furthermore, limited awareness and historical undertreatment of TR may also contribute to the low procedural volume. Notably, compared with existing studies, our analysis represents one of the larger real-world T-TEER cohorts to date, providing additional strength and generalizability to the current evidence.

Our study has several strengths, including a large sample size, contemporary real-world data, and comprehensive PSM to minimize confounding.

Nonetheless, several important limitations warrant consideration. First, as an observational database study, residual confounding cannot be completely excluded despite PSM. Although clinically established thresholds were used for variables such as BNP, BMI, and eGFR, some residual imbalance remained in the underlying continuous measurements after matching. Furthermore, important clinical variables unavailable within the TriNetX platform, including frailty measures, New York Heart Association functional class, detailed medical therapy, right ventricular function, pulmonary hemodynamics, and echocardiographic severity parameters, could not be incorporated into the analysis. Second, A key limitation is the lack of echocardiographic TR severity grading (e.g., TR severity grade, vena contracta width, effective regurgitant orifice area, leaflet tethering, TAPSE, annular dimensions) and right heart failure staging (B–D) in the TriNetX database, which precludes direct stratification by TR severity. This is inherent to large administrative datasets relying on ICD coding rather than imaging parameters. To partially mitigate this limitation, we incorporated clinically relevant indirect surrogates of disease severity, including HF status, pulmonary hypertension, and systemic congestion (e.g., edema), in the propensity score matching process. Nevertheless, residual confounding due to unmeasured TR severity likely persists, and our findings should be interpreted accordingly.

Third, detailed procedural information, including device platform, procedural success, residual TR severity, and procedure-related complications, was unavailable in the database. Consequently, we could not distinguish between dedicated tricuspid devices, such as TriClip or PASCAL, and off-label MitraClip use. Nevertheless, a recent meta-analysis by Mohamed Balata et al. showed that although TriClip was associated with a smaller vena contracta, no significant differences were observed among devices regarding effective regurgitant orifice area (EROA), regurgitant volume, or clinical outcomes (24). In addition, residual valvular dysfunction could not be adequately assessed, thereby limiting mechanistic interpretation of the observed associations.

Forth, immortal time bias and selection bias may have influenced the findings because patients in the T-TEER group were required to survive until the procedure date and likely represented a highly selected population treated at specialized centers. Although follow-up analyses were initiated after a 1-month landmark period to reduce early temporal bias, these limitations cannot be completely eliminated. Finally, coding-based definitions may have resulted in misclassification of exposures and outcomes. In addition, detailed censoring distributions, numbers-at-risk tables, and competing-risk regression analyses were not directly available within the TriNetX platform, which may limit interpretation of time-to-event analyses for nonfatal outcomes. Although follow-up analyses extended to 5 years, the relatively shorter median follow-up duration suggests that later time-point estimates should be interpreted cautiously. Future prospective randomized studies with standardized imaging assessment and detailed hemodynamic characterization are needed to confirm these observational findings and to better define the optimal timing and patient selection criteria for T-TEER intervention.

## Conclusion

In this propensity score–matched cohort, T-TEER was associated with lower all-cause mortality compared with medical therapy alone, with this association persisting up to 5 years. T-TEER was also associated with lower rates of HF exacerbations, emergency department visits, and all-cause hospitalizations during follow-up. Additionally, the procedure was linked to a more favorable composite renal outcome, suggesting a potential beneficial effect on cardiorenal interactions through congestion relief. Overall, these findings support the potential clinical value of T-TEER in patients with significant TR.

## Data Availability

The original contributions presented in the study are included in the article/supplementary material, further inquiries can be directed to the corresponding author.
